# When Is Spillover from Marine Reserves Likely to Benefit Fisheries?

**DOI:** 10.1371/journal.pone.0107032

**Published:** 2014-09-04

**Authors:** Colin D. Buxton, Klaas Hartmann, Robert Kearney, Caleb Gardner

**Affiliations:** 1 Fisheries Aquaculture and Coasts Centre, Institute for Marine and Antarctic Studies, University of Tasmania, Hobart, Tasmania, Australia; 2 Institute for Applied Ecology, University of Canberra, Canberra, Australian Capital Territory, Australia; University of Tasmania, Australia

## Abstract

The net movement of individuals from marine reserves (also known as no-take marine protected areas) to the remaining fishing grounds is known as spillover and is frequently used to promote reserves to fishers on the grounds that it will benefit fisheries. Here we consider how mismanaged a fishery must be before spillover from a reserve is able to provide a net benefit for a fishery. For our model fishery, density of the species being harvested becomes higher in the reserve than in the fished area but the reduction in the density and yield of the fished area was such that the net effect of the closure was negative, except when the fishery was mismanaged. The extent to which effort had to exceed traditional management targets before reserves led to a spillover benefit varied with rates of growth and movement of the model species. In general, for well-managed fisheries, the loss of yield from the use of reserves was less for species with greater movement and slower growth. The spillover benefit became more pronounced with increasing mis-management of the stocks remaining available to the fishery. This model-based result is consistent with the literature of field-based research where a spillover benefit from reserves has only been detected when the fishery is highly depleted, often where traditional fisheries management controls are absent. We conclude that reserves in jurisdictions with well-managed fisheries are unlikely to provide a net spillover benefit.

## Introduction

Marine reserves (MR), also known as no-take marine protected areas (MPA), are widely acknowledged as a conservation tool and their utility in a variety of situations is well established [1]. In particular over-exploited fish populations are shown to recover in the absence of fishing and generally become more abundant and attain a larger mean size in the reserve [2]. MPAs are also frequently promoted for the management of fisheries [3]–[7], even though compelling evidence in support of a net fisheries benefit is lacking [8]. Fisheries are proposed to benefit from reserves through increased production of eggs and larvae from the reserve (recruitment effect) and the net movement of adults into adjacent fishing grounds (spillover effect) [9].

In this study we focus on the spillover effect and, to avoid confusion over the use of terms, we define *spillover* as the net movement of fish across the boundary of a reserve into the fished ground, which would be expected to occur on the basis of fundamental physical principles of random movement. This is in contrast to *net spillover benefit* which involves spillover of sufficient magnitude to compensate for lost productivity due to the closure of fishing grounds, resulting in an overall benefit to the fishery through higher catch or economic yield.

Our review of the extensive literature reporting fisheries benefits reveals that there are surprisingly few empirical studies that attempt to quantify either the recruitment effect or a net spillover benefit. For example, Goni et al. [10] claims to be the first study to demonstrate a net spillover benefit in a fishery. Harrison et al. [11] make a similar claim with respect to the recruitment benefit of reserves in terms of larval export. Whilst spillover has been shown in several other studies, most do not accommodate the reduction in catch that results from reducing the area of the fishery, and consequently do not demonstrate a net spillover benefit.

Fishers are generally opposed to the introduction of reserves because they reduce the size of their fishing grounds, which is inferred to result in a loss of yield. Spillover is a common counter argument from reserve proponents, including Government agencies in the US, Europe and Australia, claiming that it will compensate for the lost fishing grounds to the extent that a net improvement in fisheries yield occurs [12]–[14].

The impact of the introduction of reserves on yield has been addressed in a number of theoretical studies (e.g., [15]–[17]), several of which progressively conclude that under broad assumptions well-managed fisheries should not benefit from the introduction of reserves [18]–[21]. Hart [22] quantifies this result to some degree by using an age-structured model, concluding that a benefit from spillover should not be anticipated unless open area fishing mortality considerably exceeds that which produces MSY.

The assumptions underlying these studies primarily concern the homogeneity of fish stocks and are reasonable for a large range of species. The obvious exception occurs in fish stocks with strong variability in spatial structure, for example where source-sink relationships exist or where reserves may result in the closure of disproportionately productive areas [23]. Such spatial heterogeneity is the basis of traditional spatial management of fisheries, and is a well-established and understood technique. Targeted spatial closures can be expected to benefit fisheries for selected species if the closed area is of disproportionate significance to the productivity of the species in question. Not surprisingly some models have shown that, at least under certain conditions, higher sustainable yields can be achieved with a marine reserve than without, e.g, [17], [23], [24]. But despite the common demonstration that special circumstances are required to achieve a spillover benefit from reserves, the implication of these findings have received limited attention and appear to have contributed little to the international public debate over fisheries benefits and to current management policy.

In this paper we use a widely applied fisheries population dynamics model which minimizes assumptions in order for the outputs to be applicable to a broad range of fisheries in non-structured environments (‘normal’ or ‘average’ fisheries). We modify this model to incorporate a MR and consider the management circumstances under which a non-specific reserve is likely to provide a benefit to the fishery. Our work highlights the effect that the degree of mismanagement under conventional fisheries management practices has on the ability of a reserve to provide a net fisheries benefit. It also investigates how this relationship changes with the rate that fish move between the reserve and the main population.

## Methods

### Population Dynamics

The population dynamics were modeled using a deterministic difference equation of the form:
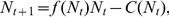
(1)where 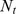 is the stock size at time 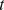, 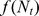 is the biological model that defines population growth and 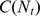 is the catch. Common examples for the biological component of this model include the Ricker model:
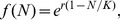
(2)and logistic model:
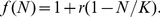
(3)In both models 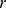 is the maximal growth rate and 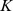 the carrying capacity (maximum population size).

Throughout this analysis we assume that the population is homogenous - a small proportion, 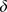, of the population will behave identically in isolation to a larger proportion of the population. Mathematically, this implies that the carrying capacity can be reduced to 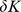. Alternatively we can consider the biological model to be a function of population density, in this case our model becomes:
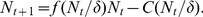
(4)


The divisor in the catch term indicates that catches are proportional to the population density (or constant).

Consider splitting a population into two areas: (i) a reserve occupying a proportion, 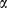, of the original habitat size and (ii) the remaining fishing grounds of size 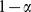. Denoting the two population sizes by 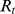 and 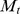 respectively, the model becomes:
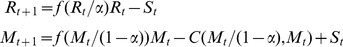
(5)where 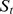 denotes the spillover from the reserve into the fished population.

### Spillover

We assume that a proportion, 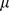, of the population in the reserve moves into the fishing ground at each time step. As the population in the reserve is 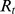, then 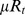 will migrate out of the reserve. Similarly a proportion, 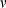, of the population in the main fishing ground will migrate into the reserve. This results in the net movement from the reserve into the main fishing ground (the spillover) being:
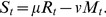
(6)


The values 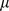 and 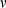 will depend on both the size and geometry of the reserve, however given the homogeneity of the population we also require that the net spillover is zero (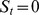) when the population density in the reserve and the fishing ground is equal (i.e. 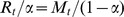). With this requirement and (6) we have:
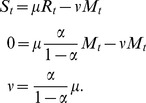
(7)


As a direct result of the assumption of spatial homogeneity, a single parameter, 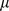, is sufficient to define the strength of the movement both in and out of the reserve. The net spillover from the reserve therefore becomes:
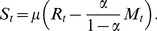
(8)Note that we assume that 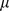 (and 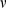) are independent of the population density in and outside of the reserve. While there may be evidence to suggest that some individuals do follow a density gradient [25], [26] this does not substantially alter our findings, as it is akin to an increase in 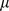.

### Fishing

We have specified the catch as a function of the population density and population size, 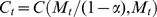. One common catch model is constant catch, as found, for example, in a subsistence fishery where a certain catch must be obtained each year to feed the population:
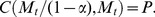
(9)


Well managed fisheries either have natural restrictions that prevent over-exploitation of the fish stock (e.g., limited demand of a niche product) or management controls to prevent over-exploitation. Management controls can be divided into two broad categories – input and output controls. Input controls limit the effort applied in the fishery. Denoting this by 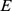 we have:
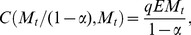
(10)where q is a constant of proportionality. With this formulation, catch is directly proportional to the effort and population density (hence division of 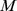 by 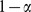 to obtain a density). Other functional forms may be more appropriate for certain fisheries and fishing methods (e.g. purse seining of schooling fish). We considered all effort applied to the fishery to shift instantaneously from the reserve to the open area.

Output controls limit the catch that can be taken from a fishery and were not explored, as the existence of an effective output control (that does not cause a fishery collapse at equilibrium) implies effective fisheries management [27]. In reality there are many examples of ineffective output controls in fisheries that have not collapsed. These fisheries persist as the output controls are adjusted through time or, when the stock is in low abundance, effort controls (whether through management or limited numbers of participating fishers) restrict the fishery. Modeling such systems requires many assumptions; hence we have focused on input controlled fisheries in this analysis.

### Net effect of the reserve on catch

We consider an effort-controlled fishery with a fish stock governed by the Logistic model. Stock size is measured in biomass, consequently growth encompasses both individual growth and recruitment. We assume that the population is homogenous and that introduction of the reserve will concentrate the effort in the remaining fishing grounds. The latter would be expected in a poorly managed fishery.

We assume that the population was at equilibrium prior to the introduction of a reserve and compare this with the post-reserve equilibrium. During the transient time between these two states spillover will be less. Since we are considering the equilibrium states we have 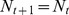 which we simply denote by 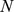, similarly for 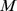, 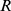 and 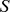.

Firstly, consider a fishery with a level of effort corresponding to near extinction, 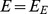. Introduction of a reserve will increase surplus production unless the population is beyond recovery.

At the other extreme, consider a pre-reserve fishery that is producing maximum sustainable yield (MSY) from the total area: 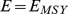. By definition at this point, surplus sustainable production cannot increase. Therefore introduction of a reserve must decrease overall catch.

At 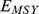 the spillover effect is less than the lost productivity and at 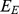 it exceeds the lost productivity. At some level of effort in between, the reserve must switch from having a net negative effect on the fishery to a net positive effect due to spillover. The level of effort at which this occurs is dependent on the model and its parameters. We now establish the point at which this occurs for a logistic model (equation (3)).

If spillover equals lost productivity in the fishing area, the pre-reserve and post-reserve catches must equal 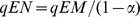; hence 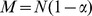. Simply put, the population density in the fishing grounds must remain unchanged. Substitution in equation (5) yields:
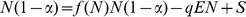
(11)subtracting equation (1) (at equilibrium) and solving for *S* gives:



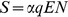
(12)Consequently, the spillover must equal the surplus production of the original fishing grounds that has now been encompassed in the reserve.

For a given level of effort, the pre-reserve fishery given by equation (4) will possess a solution, the nature of which depends on the population dynamics model. For example the non-zero solution for the logistic model is:
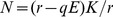
(13)


Using the full two area logistic model with effort controlled fishing (equations (5), (8) and (10)) and substituting equations (11) and (12) permits us to eliminate several of the unknowns. In this case we choose to eliminate *N, M*, *R* and *S* since conceptually we consider these to be determined by the remaining parameters. After algebraic manipulation (not shown here) we obtain the level of effort at which the introduction of the reserve does not change the overall catch:
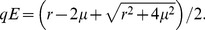
(14)Note that 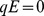 is also a solution (if no fishing is taking place, introduction of a reserve will not reduce the catch). A negative solution also exists but is of no further interest as the population would be extinct and negative densities are merely a mathematical curiosity. The same approach can be used for other population dynamics models, however for some models (e.g. the Ricker model) straight-forward analytic solutions do not exist. Qualitatively we would expect similar results for other population dynamics models and found this to be the case for numerical solutions to the Ricker model (results not shown here).

The optimal effort for this fishery without a reserve is 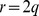. We divide equation (14) by this and subtract 1 to obtain the minimum excess effort (as a proportion) required for a reserve to be beneficial:
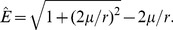
(15)This depends only on the ratio of the movement rate out of the reserve to the growth rate of the stock (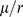), and not on the proportion of the area dedicated to the reserve (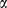). However, it should be noted that the movement rate out of the reserve, 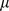, is likely to depend on the reserve size. This link has not been explicitly explored here, however, for a given choice of 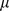, there is likely to be only a limited range of values of 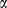 that is possible.

Equations (14) and (15) are derived in more detail in Appendix S1.

## Results

**Figure 1:**
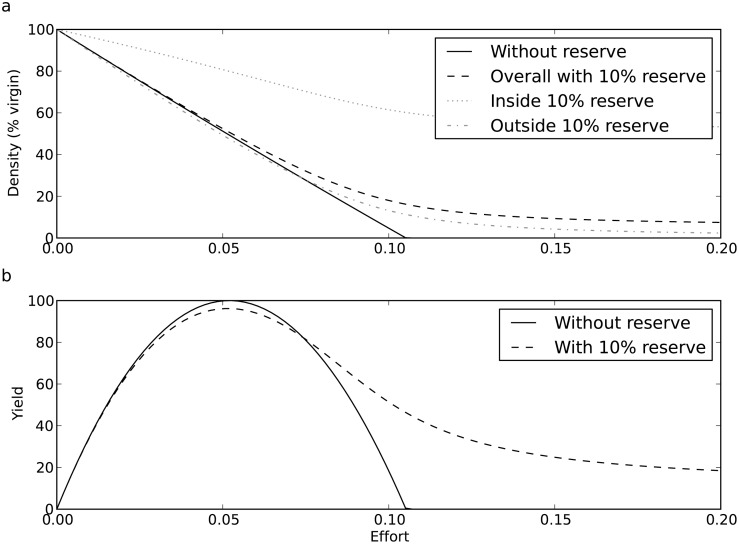
Changes in population and fishery dynamics resulting from the introduction of a reserve. (a) The equilibrium biomass density as a function of fishing effort. The density is shown for the whole stock without a reserve and with a 10% reserve. For the reserve scenario the density inside and outside of the reserve is also shown. (b) Yield as a function of fishing effort both with and without a reserve.

**Figure 1** shows an example where a 10% reserve is introduced with 5% movement out of the reserve (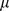) and a maximum growth rate (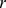) of 10%. This figure explores the effect of a reserve for different levels of initial effort applied to the fishery. The maximum sustainable yield (MSY) is obtained with an effort of 0.05 (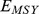).

Introduction of the reserve decreases the yield at 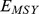 and by definition there is no alternative effort that produces the same maximal yet sustainable yield. The point of intersection in the bottom panel corresponds to a level of effort, 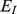, where the yield is the same with or without a reserve. At levels of effort above 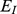, the introduction of a reserve increases yield. In this scenario, 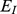 is 150% of 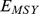, so a fishery would have to have 50% excess effort for the reserve to be beneficial in terms of the yield of the target species. At even higher levels of effort (>150% 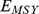) the MPA mitigates the impact of overfishing and permits sustainable (but substantially reduced) yield.

**Figure 2:**
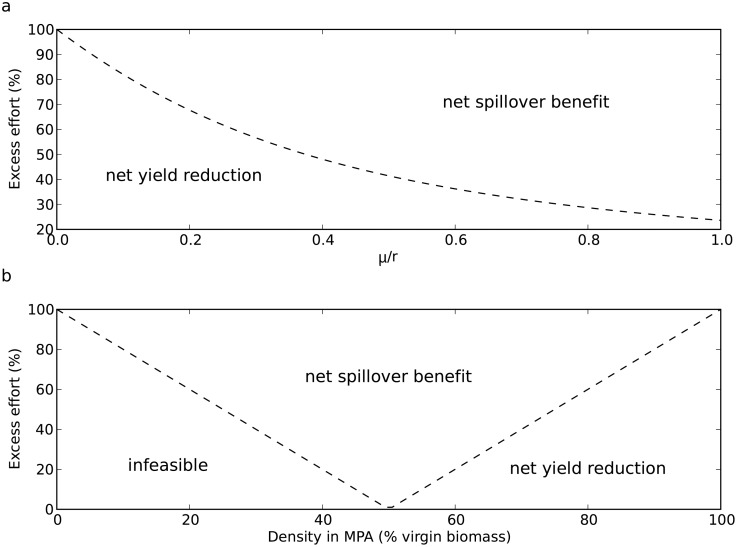
Characterisation the management and biological circumstances in which a reserve is beneficial. (a) The excess effort required for a reserve to improve fishery yield. For our simple model this was found to depend only on the ratio of the movement rate out of the reserve (and thus on reserve size) to the growth rate of the stock (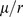). (b) The excess effort required for optimality as a function of the reserve density (at equilibrium). For example a reserve with 80% virgin biomass at equilibrium will provide a net economic benefit for a fishery that has more than 60% excess effort relative to optimal management. Combinations of excess effort and reserve density that fall in the bottom left region are infeasible; in these situations a reserve would have to decrease in population density after being formed (not possible in our model). Inside the “V” the reserve provides a net increase in fishery yield. In the right region the reserve decreases yield.

The level of excess effort at which a reserve has a neutral impact on fisheries yield depends only on the ratio of movement out of the reserve (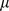) to the maximum growth rate (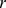) (equation (15)). This relationship is shown in Figure 2a, when the movement rate is high relative to the growth rate, a reserve is beneficial at low levels of excess effort. The extreme situation where 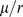 approaches infinity corresponds for example to a miniscule reserve, which clearly will have negligible impact on a fishery. At the other extreme, 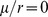, there is no movement out of the reserve, consequently it will always have a negative impact.

Alternatively we consider the excess effort required for a reserve to be beneficial as a function of the reserve density at equilibrium (Figure 2b). If the reserve is at 50% virgin biomass density it has neutral effect on the fishery. This is because 50% virgin biomass corresponds to MSY in this model and all surplus production is moved to the main population through spillover. At reserve densities above this, a fishery must have more excess effort to benefit from a reserve. In particular if reserves have a high percentage of virgin biomass (a common conservation goal for reserves) they will only benefit fisheries that have greater mismanagement. For example, at 80% virgin biomass a reserve will only benefit fisheries with more than 60% excess effort.

## Discussion

### Model outcomes

The model presented here examines the circumstances under which spillover from a reserve is sufficient to increase fishery yield (thus providing a net spillover benefit). As expected, density of exploited species was higher in the reserve than the fished area, which may be mistaken in itself as evidence that the reserve will create a net beneficial increase through larvae production [28]. However, it is important to consider the net effect, which in our model case was a decline in average density and a loss of yield except where effort exceeded 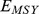. While models are by necessity a simplification of ecological complexity, we show that the extent to which effort must exceed 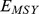 for any yield benefit to occur from the reserve depends on the ratio of the rate of movement out of the reserve and the growth rate of the species concerned. Highly mobile/slow growing species received relatively less benefit from reserves where effort was above management targets compared to species with low movement/fast growth.

Our model is a relatively simple one chosen to illustrate a fundamental principle that is applicable across a broad range of fisheries. Different formulations for the biological model, 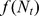, can be specified and similar results were obtained for the Ricker model (not shown here). Three major assumptions were made to maintain model simplicity: spatial homogeneity, density dependence and steady state dynamics.

*Spatial homogeneity* is an inappropriate assumption for some species. For example, where there are clear source-sink relationships protecting the source in a reserve is likely to provide an overall benefit [29]. The location of source areas can be consistent across different species and trophic levels, and in rare cases where these locations are known, it becomes possible to locate reserves that provide benefit to numerous, and theoretically all, species [30].

*Density dependence* in our model is a function of the total biomass in the local area (i.e. the fished population or the reserve population). This does not adequately capture the dynamics of species where density dependence varies substantially with age (e.g. density dependence occurring primarily during larval stages) and where different age classes have different movement rates across the reserve boundary. In such situations it could be possible for the reserve to provide a greater benefit by providing a recruitment increase to the fished region.

*Steady state dynamics* are widely used to explore fundamental fisheries principles. In the context of reserves, some models have shown that biological stochasticity may lead to theoretical net spillover benefits in fisheries where the biomass can be determined accurately on an annual basis and corresponding perfect catch limits set each year [31], [32]. Given the unrealistic nature of this assumption for most management situations there would be some value in further research that explored reserve benefits in a stochastic setting with realistic management. After the introduction of a reserve, it will take some time for the reserve population to build to the final density. Consequently it is expected that the reduction in yield will initially be much greater than predicted by our steady state model. With the concentration of effort the fished population would initially decrease before increasing some time later due to spillover from the reserve.

Our model did not consider that the introduction of a reserve may result in an effort reduction due, for example, to decreased accessibility or increased fishing costs. This would be beneficial for stock status and overall production in over-exploited fisheries, however, it would result in a reduction of production in well-managed fisheries.

Under our model there were no combinations of growth rate or movement where a net spillover benefit from reserves could occur unless effort exceeded 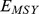. Where effort is less than 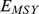, a loss of yield always occurs when reserves are implemented. The level of excess effort beyond 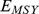 at which a reserve provides net spillover benefits was shown to depend only on the ratio of movement out of the reserve to the rate of growth of the population (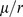). We also showed that reserve configurations that achieve higher densities of stock are only beneficial for mismanaged fisheries (Figure 2b). For example, a reserve that ultimately increases biomass density to 75% of unfished levels would benefit a fishery if the initial effort exceeds 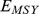 by more than 50%. These results show that reserves will generally negatively impact yield for well managed fisheries. However reserves could minimize their impact on a well managed fishery by reducing the density increase of the fishery’s target species in the reserve. For example, a reserve could be of a sufficient size to protect species with small home ranges whilst being small enough that individuals of the target species frequently move beyond reserve boundaries (a high movement rate, 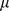). This could also be achieved by having high reserve boundary length to total area ratios. The feasibility of this outcome will depend on the movement characteristics of the species involved.

Our finding that reserves cannot improve the yield of a well-managed fishery is consistent with several other theoretical studies [18]–[20]. The work here extends these findings by exploring the extent to which a fishery must be mismanaged before introduction of a reserve provides a benefit to the fishery in terms of yield.

Many fisheries have management objectives that constrain catch below the target of MSY assumed here, for example to manage risk from stochastic processes such as recruitment, or where there is an objective to target a maximum economic yield (MEY) that is variant to MSY. In these fisheries, effort and catch are lower than would occur with the MSY target [33], which reduces the negative impact of reserves on total yield, but also shifts the fishery further away from the level of depletion required for a net spillover benefit to occur.

### Empirical context

The results from this study are consistent with other studies that have modeled the impact and/or benefits of reserves on fisheries in terms of improvements in yield. Following the publication of the early models on the potential net spillover benefits from reserves [16], [18], [34] there have been surprisingly few empirical studies that have attempted to demonstrate the effect. Most of the reserve literature has concentrated on the changes within reserves, showing an increase in size and abundance of resident fish and crustaceans, particularly of reef associated species (for a review see [35]). Despite the lack of empirical evidence the argument persists that reserves will confer a net spillover benefit to fisheries [5]. This view is actively promoted by government agencies [12]–[14]. However, the literature confirms that the evidence for such a benefit is far from conclusive. Several studies report a lack of evidence for spillover due to the low movement at the scale of the reserve [36]–[38], while others showed that spillover occurred but not that lost yield was compensated to produce a net benefit (e.g., [39]–[41]).

While density dependent export from reserves is considered to be a rational expectation [42], no studies have been able to conclusively demonstrate a net spillover benefit, and leakage from reserves is probably more related to random movement within species (e.g., [38], [41], [43], [44]). Several studies fail to provide conclusive evidence for net spillover benefits, yet argue that reserves are needed to provide fishery benefits (e.g., [39], [40]). Spillover has been inferred from observations of a density gradient between the reserve and adjacent fished area (e.g., [45], [46]) even though evidence was acknowledged to be equivocal (e.g., [42], [47], [48]), and where confounding factors such as a change in fishing practices (e.g., [49]) or changed fisheries management strategies over the study period were ignored (e.g., [4], [7]). Few of these studies consider whether the purported spillover to the fishery (as inferred from catch rates) has actually resulted in a net spillover benefit for the fishery. Even if CPUE goes up in a fished area it may be insufficient to result in a net production gain for the whole of the fishery.

Several studies have been able to demonstrate that spillover has contributed to an improvement in biomass and thus catch rate adjacent to the reserve [50]–[53]. These examples, all in the Mediterranean, were conducted in areas where the total fishery had been severely depleted. In this respect they are similar to several studies in other areas that, on multiple lines of evidence, infer a net spillover benefit to fisheries. Examples come from Africa [48] and Asia [7], [42], [47] where the fisheries in question were over-exploited and where there was limited application and/or enforcement of standard fisheries management controls. The result was that the proclamation of a reserve resulted in a recovery of the population in the reserve and a subsequent improvement in catches close to the reserve boundary. This is consistent with our conclusion that reserves can provide a net spillover benefit for severely depleted stocks. It does not, however, provide evidence that the declaration of the reserve was the most efficient means of achieving that benefit.

There are many possible variations on the biological assumptions made in our model. Aspects such as stock heterogeneity and variant density dependence assumptions will influence the impacts of a reserve as well as the level of mismanagement, where a reserve switches from being beneficial to being detrimental for a fishery.

The model results presented here are for a general case, which is appropriate for consideration of reserves where a large number of species with variable life histories and spatial distributions are affected by change in management. Closed areas for traditional fishery management purposes are applied on a species by species basis and may have very different management outcomes to reserves because they can be designed and located to affect an individual stock. There are numerous cases where species with spatial heterogeneity, such as spawning aggregations or larval source-sink dynamics, benefit from fishery closures that target important source areas [54]. A total fishing closure would achieve the same result for those species, but can be expected to have less beneficial results for other exploited species.

### Conclusions

We conclude that in fisheries where there is effective management, marine reserves are unlikely to produce a net spillover benefit for the total fishery, whereas they may be beneficial where the fishery has been mismanaged and stocks severely depleted. These results expand the implications of previous work by providing estimation and evaluation of the degree of mismanagement of fisheries that is necessary for non-specific closures to provide net benefits to fisheries.

The conclusions from the modeling presented here are supported by review of empirical studies, where spillover benefits have only been conclusively demonstrated in highly depleted areas. Together with the combined weight of earlier modeling work, they suggest that a net benefit from spillover should not be expected in areas already benefiting from quality traditional fisheries management.

These generalised findings in relation to reserves should not be confused with the use of targeted spatial closures for single fisheries, where it is possible to increase yield through closures by taking account of the spatial heterogeneity of life history traits.

While reserves may be proclaimed for a range of conservation objectives (including addressing impacts such as the effect of fishing on benthic environments, interactions with threatened species and catch of non-target species), we contend that it is misleading for governments to promote reserves on the basis of net spillover benefit in the context of well-managed fisheries. Reserves are only likely to be an effective strategy for fisheries management where effort is not or cannot be effectively controlled across the wider stock.

## Supporting Information

Appendix S1 **Detailed derivation of Logistic MPA equilibrium.**
(DOCX)
