# Supplementary material for: When Is Spillover from Marine Reserves Likely to Benefit Fisheries?
Source: PLoS One. 2014 Sep 4;9(9):e107032. doi: 10.1371/journal.pone.0107032 (PMC4154848; doi:10.1371/journal.pone.0107032)
Supplement: Appendix S1 — Detailed derivation of Logistic MPA equilibrium. (DOCX) [file pone.0107032.s001.docx]

# Appendix S1

# Detailed derivation of Logistic MPA equilibrium

This section provides a detailed derivation of equations (14) and (15) which characterize the level of effort at which introduction of a reserve provides no change to the fishing catch.

To determine whether the reserve introduces a positive change to the fishery it is first necessary to consider the dynamics without a reserve. This establishes what optimal management would have been without a reserve and what catch this would have produced.

## Population dynamics without a reserve

The population dynamics for the fished area without a reserve is a classic model that has been broadly studied and is detailed in many introductory mathematical ecology textbooks. The model is produced from our generalized single area model (equation (4)) by substituting the equation for fishing dynamics (equation (10)) and population dynamics (equation (3)):

$$N_{t+1}=\left[1+r\left(1-\frac{N_{t}}{K}\right)\right]N_{t}-qEN_{t},$$

at equilibrium we have $N_{t+1}=N_{t}$ and this becomes:

$$N=\left[1+r\left(1-\frac{N}{K}\right)\right]N-qEN.$$

The non-zero solution of this is readily solved to give:

$$N=K\left(1-\frac{qE}{r}\right)$$

The equilibrium catch is:

$$C=qEN$$

$$C=qEK\left(1-\frac{qE}{r}\right).$$

This is maximized when:

$$E=r/2q,$$

which corresponds to the maximum sustainable yield (MSY).

## Population dynamics with a reserve

The population dynamics of the two areas found by substituting the equation for spillover (equation (8)), fishing dynamics (equation (10)) and population dynamics (equation (3)) in the generalized two area model (equation (5)):

$$R_{t+1}=\left[1+r\left(1-\frac{R_{t}}{\alpha K}\right)\right]R_{t}-\mu\left(R_{t}-\frac{\alpha}{1-\alpha}M_{t}\right)$$

$$M_{t+1}=\left[1+r\left(1-\frac{M_{t}}{(1-\alpha)K}\right)\right]M_{t}-\frac{qEM_{t}}{1-\alpha}+\mu\left(R_{t}-\frac{\alpha}{1-\alpha}M_{t}\right).$$

To find the equilibrium solution we have $M_{t+1}=M_{t}$ and $R_{t+1}=R_{t}$ so the above becomes:

$$R=\left[1+r\left(1-\frac{R}{\alpha K}\right)\right]R-\mu\left(R-\frac{\alpha}{1-\alpha}M\right)$$

$$M=\left[1+r\left(1-\frac{M}{(1-\alpha)K}\right)\right]M-\frac{qEM}{1-\alpha}+\mu\left(R-\frac{\alpha}{1-\alpha}M\right).$$

For the introduction of this reserve to have no net impact on the catch, the catch from the pre-reserve fishery must equal the catch from the post-reserve fishery:

$$qEN=\frac{qEM}{1-\alpha}$$

$M=N(1-\alpha)$ .

Substitution in equation yields:

$$N(1-\alpha)=\left[1+r\left(1-\frac{N(1-\alpha)}{(1-\alpha)K}\right)\right]N(1-\alpha)-\frac{qEN(1-\alpha)}{1-\alpha}+\mu\left(R-\frac{\alpha}{1-\alpha}N(1-\alpha)\right)$$

$$N(1-\alpha)=\left[1+r\left(1-\frac{N}{K}\right)\right]N(1-\alpha)-qEN+\mu\left(R-\alpha N\right)$$

Subtracting equation from $1-\alpha$ times equation gives:

$$0=0+qEN-(1-\alpha)qEN-\mu\left(R-\alpha N\right)$$

$$\alpha qEN=\mu\left(R-\alpha N\right)$$

As noted in equation (12) in the main manuscript, this implies that the spillover must equal the surplus production of the original fishing grounds that has now been encompassed in the reserve.

This can be re-arranged to give $R$ as a function of $N$:

$$R=\frac{\alpha qEN}{\mu}+\alpha N$$

Substitution of equation in equation gives:

$$R=\left[1+r\left(1-\frac{R}{\alpha K}\right)\right]R-\alpha qEN$$

$$0=r\left(1-\frac{R}{\alpha K}\right)R-\alpha qEN$$

Substitution of equations and in equation after simplification yields:

$$\begin{matrix} 0=\mu^{2}r\left(\frac{1}{\mu}\left[1+\frac{\mu qE-\mu r+q^{2}E^{2}-qEr}{\mu r}\right]\left[\mu qE-\mu r+q^{2}E^{2}-qEr\right]-qE\left[1-\frac{qE}{r}\right]\right) \\ 0=\mu^{2}(qE-r)-(\mu+qE-r)\left(\mu qE-\mu r+q^{2}E^{2}-qEr\right). \end{matrix}$$

A cubic in $qE$ which has a real, positive solution:

$$qE=\left(r-2\mu+\sqrt{r^{2}+4\mu^{2}}\right)/2$$

This is the level of effort at which a reserve does not alter the catch and is equal to equation (14) in the main paper. The relative level of excess effort that this corresponds to is found by dividing by the optimal effort, equation , and subtracting 1, yielding:

$$\hat{E}=\sqrt{1+(2\mu/r)^{2}}-2\mu/r,$$

equation (15) in the main paper.
